# Post‐endoscopic retrograde cholangiopancreatography pancreatitis in single‐stage endoscopic common bile duct stone removal

**DOI:** 10.1002/jgh3.12263

**Published:** 2019-10-15

**Authors:** Hirokazu Saito, Takehiko Koga, Masafumi Sakaguchi, Yoshihiro Kadono, Kentaro Kamikawa, Atsushi Urata, Haruo Imamura, Shuji Tada, Tatsuyuki Kakuma, Ikuo Matsushita

**Affiliations:** ^1^ Department of Gastroenterology Kumamoto Chuo Hospital Kumamoto City Kumamoto Japan; ^2^ Department of Gastroenterology Saiseikai Kumamoto Hospital Kumamoto City Kumamoto Japan; ^3^ Department of Gastroenterology Kumamoto City Hospital Kumamoto City Kumamoto Japan; ^4^ Department of Biostatics Center, Medical School Kurume University Kurume City Fukuoka Japan

**Keywords:** common bile duct stone, ERCP, post‐ERCP pancreatitis, single‐stage endoscopic stone removal

## Abstract

**Background and Aims:**

Papillary treatment, such as endoscopic sphincterotomy or endoscopic papillary balloon dilation, and subsequent single‐stage endoscopic stone removal are often performed for choledocholithiasis; however, the incidence of postendoscopic retrograde cholangiopancreatography pancreatitis (PEP) is unclear. This study aimed to compare the overall incidence of PEP between single‐ and two‐stage stone removal groups and the incidence of PEP between these two groups based on cannulation time.

**Methods:**

We included 897 patients with native papilla who underwent papillary treatment and stone removal for choledocholithiasis with no inflammation or mild‐to‐moderate acute cholangitis at three institutions between April 2012 and March 2018 in Japan. We performed a propensity‐matched analysis and regression adjustment by propensity score to adjust for potential confounding factors.

**Results:**

In the propensity‐matched analysis with 234 pairs, there was no significant difference in the overall incidence of PEP between single‐ and two‐stage stone removal procedures (15/234, 6.4% *vs* 6/234, 2.6%, *P* = 0.072, respectively). Although single‐stage stone removal after a cannulation time of ≤15 min was not a significant risk factor of PEP, the procedure after a cannulation time of >15 min was a significant risk factor of PEP as estimated by regression adjustment by propensity score (*P* = 0.014, 95% CI = 1.4–19.4, odds ratio = 5.2).

**Conclusions:**

Single‐stage endoscopic stone removal after a long cannulation time of >15 min increased the incidence of PEP compared with the two‐stage stone removal procedure. Thus, single‐stage stone removal should be performed in patients with a cannulation time ≤ 15 min.

## Graphical abstract

Papillary treatment and subsequent single‐stage endoscopic stone removal are often performed for choledocholithiasis. However, the incidence of post‐endoscopic retrograde cholangiopancreatography pancreatitis (PEP) is unclear. This study demonstrated that single‐stage endoscopic stone removal after a long cannulation time of >15 min increased the incidence of PEP compared with the two‐stage stone removal procedure.

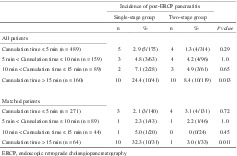

## Introduction

Stone removal by endoscopic retrograde cholangiopancreatography (ERCP) is an effective strategy for common bile duct (CBD) stones.^1^, ^2^, ^3^ Endoscopic stone removal is completed following endoscopic sphincterotomy (EST) and endoscopic papillary balloon dilation (EPBD) using a basket or balloon in 85–90% of patients with CBD stones. For large CBD stones, mechanical lithotripsy has a high success rate for stone removal.^1^ Endoscopic papillary large balloon dilation (EPLBD) is effective for the removal of multiple and large CBD stones that are difficult to remove by EPBD or EST alone.^4^

In the Tokyo guidelines 2018, papillary treatments, such as EST or EPBD, and subsequent single‐stage stone removal are acceptable in patients with mild‐to‐moderate acute cholangitis.^5^ Therefore, single‐stage endoscopic stone removal is often performed for choledocholithiasis in clinical practice.

However, ERCP is a high‐risk procedure with complications, such as cholangitis, bleeding, perforation, and pancreatitis. Of these complications, post‐ERCP pancreatitis (PEP) is the most common and serious complication, which can be lethal.^6^ Although endoscopic stone removal requires papillary treatment, including EST, EPBD, or EPLBD, these papillary treatments are risk factors of PEP.^4^, ^7^ One concern is that the risk of PEP may increase when single‐stage endoscopic stone removal is performed in addition to papillary treatment at the same time because of exacerbation of papilla trauma, especially in cases of prolonged biliary cannulation. On the other hand, single‐stage endoscopic stone removal has some advantages, such as shortened hospital stays and reduced ERCP attempts, which lead to reduced medical costs.^8^ However, there are few reports on the incidence of PEP in single‐stage endoscopic stone removal in patients with native papilla.

The aim of this study was to compare the overall incidence of PEP between single‐stage and two‐stage stone removal procedures and the incidence of PEP between these two procedures based on cannulation time for choledocholithiasis, with no inflammation or mild‐to‐moderate acute cholangitis, in patients with native papilla.

## Materials and methods

### *Patient selection and data source*

Using medical records between April 2012 and March 2018, we identified 1244 patients who had CBD stone diseases with native papilla and normal gastrointestinal tract or Billroth I gastrectomy and who underwent papillary treatment, including EST, EPBD, and EPLBD, at Kumamoto Chuo Hospital, Kumamoto City Hospital, and Saiseikai Kumamoto Hospital. Of these patients, we excluded those who had biliary pancreatitis (*n* = 113), severe acute cholangitis (*n* = 81), undetected CBD stones during ERCP (*n* = 101), unsuccessful stone removal (*n* = 37), and unsuccessful deep cannulation (*n* = 15). Finally, 897 patients with mild‐to‐moderate acute cholangitis, obstructive jaundice, elevated liver test results, and asymptomatic CBD stones were analyzed. Informed consent was obtained from all the patients. The Institutional Review Boards of each institution approved this study, and it was performed in accordance with the Declaration of Helsinki.

### *Diagnostic procedure*

In this study, one or more imaging examinations, including computed tomography, magnetic resonance cholangiopancreatography, ultrasonography, endoscopic ultrasonography, and cholangiography from percutaneous transhepatic gallbladder drainage tube, were performed to diagnose the presence of CBD stones in all patients. In patients with undetected CBD stone by imaging, we diagnosed CBD stones on the basis of clinical diagnosis, such as abdominal pain and/or elevated liver test results and/or dilated CBD. For diagnosis and grading of acute cholangitis, we used the Tokyo guidelines 2018.^9^

### *Therapeutic procedure*

#### *Premedication, scopes, and devices for stone removal*

Midazolam and pethidine hydrochloride were used for sedation, and scopolamine butylbromide, or glucagon was used for duodenal relaxation. We used side‐viewing duodenoscopy (Olympus JF‐260, TJF‐260V; Olympus Medical Systems, Tokyo, Japan) in all the patients. A basket and/or a balloon catheter and/or a mechanical lithotriptor were used to remove CBD stones.

#### *Endoscopists*

Of a total of 29 different endoscopists, 11 were trainees, defined as endoscopists who had experienced <200 ERCP procedures or who could complete ERCP that corresponded to grade 1 based on the grading scale of the ERCP core curriculum.^10^

### *Definitions*

#### *Single‐stage and two‐stage endoscopic stone removal*

Single‐stage endoscopic stone removal was defined as CBD stone removal in addition to papillary treatment, including EST, EPBD, or EPLBD at the same time. Two‐stage endoscopic stone removal was defined as CBD stone removal a few days after papillary treatment and subsequent biliary drainage.

#### *Difficult deep cannulation*

We defined difficult cannulation as cases requiring >10 min for deep cannulation in this study because a cannulation attempt requiring >10 min has been previously found to be a definite risk factor of PEP.^7^

#### *Post‐ERCP pancreatitis*

PEP was diagnosed and graded on the basis of the consensus criteria by Cotton *et al*.^11^ The diagnostic criteria of PEP were new or worsening abdominal pain with serum amylase >3 times the normal at 24 h after ERCP requiring new or prolonged hospitalization of ≥1 night. Mild PEP was defined as cases requiring hospitalization for 2–3 days, and moderate PEP was defined as cases requiring hospitalization for 4–10 days. Severe PEP was defined as cases requiring hospitalization for >10 days or percutaneous drainage or surgery.

### *Outcomes*

Primary outcomes of this study were the overall incidence of PEP for single‐stage stone removal procedure and the incidence of PEP for this procedure based on cannulation time. Secondary outcomes were the mean number of ERCP sessions for stone clearance and mean length of hospital stay.

### *Statistical analysis*

The patients' characteristics and clinical risk factors of PEP were compared between the single‐stage stone removal group and two‐stage stone removal group by using chi‐square tests, Fisher's exact tests, or *t*‐tests as appropriate. The association between single‐stage stone removal and the overall incidence of PEP was examined by performing one‐to‐one propensity score matching with a caliper and a standard deviation of 0.2 and regression adjustment by propensity score to adjust for potential confounders. Each characteristic variable of the patient and the clinical risk factors of PEP shown in Tables 1 and 2 were used to construct propensity scores.^12^ C‐statistics were calculated to evaluate the goodness of fit. We used standardized mean difference (SMD), which is not influenced by sample size, to check the balance of matched samples between single‐ and two‐stage stone removal groups. Propensity‐matched samples with SMD <0.10 is desirable.^13^ In this study, SMD in almost all factors shown in Tables 1 and 2 was <0.10, and SMD in only a factor of pancreatic injection was 0.105. Regression adjustment by propensity score was used to examine the association between the incidence of PEP and single‐stage stone removal procedure based on the cannulation time. In this analysis, we constructed propensity scores for each of the four groups of single‐stage stone removal procedure after the cannulation times of ≤5, >5 to ≤10, >10 to ≤15, and >15 min. Thereafter, a logistic regression analysis was performed in each of the four groups, where the incidence of PEP took place of the outcome, and the binary variable of the single‐ and two‐stage stone removal procedure groups and the estimated propensity scores took the place of explanatory variables. A *P* value <0.05 was used as the threshold for statistical significance. JMP® Pro 13 (SAS Institute, Cary, NC, USA) and R version 3.5.1 (http://www.r-project.org) were used to perform all the statistical analyses.

**Table 1:** Characteristics of all patients and matched patients in the single‐stage and two‐stage stone removal groups

	All patients (*n* = 897)					Matched patients (*n* = 468)				
	Single‐stage group		Two‐stage group			Single‐stage group		Two‐stage group		
	(*n* = 307)		(*n* = 590)			(*n* = 234)		(*n* = 234)		
	*n*	%	*n*	%	*P value*	*n*	%	*n*	%	*P* value
Age (mean [SD], years)	70.6 (14.2)		74.5 (13.7)		<0.001	71.9 (13.8)		70.7 (15.0)		0.36
Gender (female)	144	46.9	267	45.3	0.69	106	45.3	114	48.7	0.52
Indication for ERCP					<0.001					0.89
Mild cholangitis	94	30.6	215	36.4		85	36.3	86	36.8	
Moderate cholangitis	36	11.7	170	28.8		35	15.0	33	14.1	
Obstructive jaundice and/or elevated liver test results without cholangitis	108	35.2	137	23.3		73	31.2	79	33.8	
Asymptomatic CBD stones	69	22.5	68	11.5		41	17.5	36	15.4	
Comorbidities					0.19					0.99
Acute pancreatitis	3	1.0	1	0.2		1	0.4	1	0.4	
Chronic pancreatitis	0	0	1	0.2		0	0	0	0	
Hemodialysis	3	1.0	7	1.2		3	1.3	4	1.7	
Cardiovascular disease	28	9.1	76	12.9		26	11.1	22	9.4	
Cerebrovascular disease	15	4.9	47	8.0		14	6.0	12	5.1	
Liver cirrhosis	2	0.7	8	1.4		2	0.9	3	1.3	
Diabetes mellitus	38	12.4	71	12.0		29	12.4	31	13.2	
≥2 comorbidities	33	10.7	60	10.2		24	10.3	23	9.8	
Periampullary diverticulum	92	30.0	153	25.9	0.23	68	29.1	65	27.8	0.84
Multiple stones	122	39.7	225	38.1	0.69	91	38.9	87	37.2	0.78
Large stone (>10 mm)	33	10.7	113	19.2	0.002	29	12.4	30	12.8	1.0
Gallbladder stones	217	70.7	343	58.1	<0.001	156	66.7	163	69.7	0.55
Postcholecystectomy	27	8.8	73	12.4	0.13	22	9.4	19	8.1	0.74
Performance status (3 or 4)	40	13.0	91	15.4	0.39	37	15.8	32	13.7	0.60

CBD, common bile duct; ERCP, endoscopic retrograde cholangiopancreatography.

**Table 2:** Clinical risk factors of post‐ERCP pancreatitis in all and matched patients between the single‐stage and two‐stage stone removal groups

	All patients					Matched patients				
	Single‐stage group		Two‐stage group			Single‐stage group		Two‐stage group		
	(*n* = 307)		(*n* = 590)			(*n* = 234)		(*n* = 234)		
	*n*	%	*n*	%	*P* value	*n*	%	*n*	%	*P* value
Normal serum bilirubin	153	49.8	193	32.7	<0.001	105	44.9	98	41.9	0.58
Nondilated common bile duct	184	59.9	263	44.6	<0.001	127	54.3	131	56.0	0.78
Pharmacological prevention	161	52.4	272	46.1	0.083	117	50.0	113	48.3	0.78
Trainee endoscopist	63	20.5	98	16.6	0.17	44	18.8	40	17.1	0.72
Pancreatic injections	115	37.5	302	51.2	<0.001	88	37.6	100	42.7	0.30
PGW‐assist cannulation	31	10.1	127	21.5	<0.001	26	11.1	33	14.1	0.40
Precut sphincterotomy	5	1.6	36	6.1	0.004	5	2.1	6	2.6	1.0
EPBD or EPLBD	42	13.7	60	10.2	0.14	26	11.1	27	11.5	1.0
Cannulation time					0.043					0.84
≤5 min	175	57.0	314	53.2		140	59.8	131	56.0	
>5 to ≤10 min	63	20.5	96	16.3		43	18.4	46	19.7	
>10 to ≤15 min	28	9.1	61	10.3		20	8.5	24	10.3	
>15 min	41	13.4	119	20.2		31	13.2	33	14.1	
Absence of pancreatic stent placement	296	96.4	498	84.4	<0.001	224	95.7	221	94.4	0.67
Use of ≥2 devices for stone removal	136	44.3	344	58.3	<0.001	112	47.9	105	44.9	0.58
Procedure time (SD)	29.5 (15.4)		25.9 (15.3)		<0.001	27.5(13.7)		28.1 (17.9)		0.70

EPBD, endoscopic papillary balloon dilation; EPLBD, endoscopic papillary large balloon dilation; ERCP, endoscopic retrograde cholangiopancreatography; PGW, pancreatic guide wire.

## Results

### *Patients' demographics and clinical risk factors*

Patients' demographics and clinical risk factors of all patients and propensity‐matched patients who underwent single‐stage and two‐stage stone removal are shown in Tables 1 and 2, respectively. Pharmacological prevention included the use of a protease inhibitor and rectal nonsteroidal anti‐inflammatory drugs (NSAIDs). There was no significant difference in the incidence of PEP between a protease inhibitor and rectal NSAIDs in all patients and matched patients (*P* = 0.55 and *P* = 1.0, respectively).

### *Successful cannulation rates*

The successful cannulation rate in patients diagnosed as having CBD stone diseases, excluding biliary pancreatitis and severe acute cholangitis, was 1035 of 1050 (98.6%). Fifteen patients with unsuccessful cannulation were excluded in the present study.

### *Rate of complete stone clearance*

The rate of CBD stones detected by ERCP in patients diagnosed as having CBD stones on the basis of imaging or clinical findings, excluding biliary pancreatitis, severe acute cholangitis, and unsuccessful cannulation, was 934 of 1035 (90.2%). The 101 patients in whom CBD stones were not detected by ERCP were excluded from this study. The rate of complete stone clearance was 897 of 934 (96.0%). In addition, the 37 patients with unsuccessful stone removal were excluded.

### *Incidence rates of PEP*

Table 3 presents the incidences of PEP in single‐stage stone removal and two‐stage stone removal. In all patients, of 307 patients with single‐stage stone removal, PEP occurred in 20 (6.5%) patients, and of 590 patients with two‐stage stone removal, PEP occurred in 21 (3.6%) patients. There was no significant difference in the incidence of PEP between the single‐stage and two‐stage stone removal groups (*P* = 0.062). In propensity‐matched patients with 234 pairs, the incidences of PEP were 15 (6.4%) patients in the single‐stage stone removal group and 6 (2.6%) patients in the two‐stage stone removal group. There were no significant differences in the incidence of PEP in the matched patients between the two groups (*P* = 0.072; Table 3).

**Table 3:** Incidences of post‐ERCP pancreatitis in all patients and matched patients

	All patients					Matched patients				
	Single‐stage group		Two‐stage group			Single‐stage group		Two‐stage group		
	(*n* = 307)		(*n* = 590)			(*n* = 234)		(*n* = 234)		
	*n*	%	*n*	%	*P* value	*n*	%	*n*	%	*P* value
Post‐ERCP pancreatitis (+)	20	6.5	21	3.6	0.062	15	6.4	6	2.6	0.072
Post‐ERCP pancreatitis (−)	287	93.5	569	96.4		219	93.6	228	97.4	

ERCP, endoscopic retrograde cholangiopancreatography.

### *Effect of single‐stage stone removal for the overall incidence of PEP after adjusting for confounding factors*

We performed propensity score analysis, including propensity‐matched analysis and regression adjustment by propensity score, to adjust for confounding factors. Table 4 shows the results of the propensity score analysis. Single‐stage stone removal was not a significant risk factor in propensity‐matched analysis and regression adjustment for confounding factors by propensity score (*P* = 0.072, 95% CI = 0.99–6.8 and *P* = 0.094, 95% CI = 0.90–3.8, respectively).

**Table 4:** Effect of single‐stage stone removal for post‐ERCP pancreatitis after adjusting for confounding factors

Statistical analysis method	*P* value	95% CI
Propensity‐matched analysis	0.072	0.99–6.8
Regression adjustment by propensity score	0.094	0.90–3.8

ERCP, endoscopic retrograde cholangiopancreatography.

### *Cannulation time‐based comparison of the association between the incidence of PEP and single‐ and two‐stage stone removal procedures*

In all and propensity‐matched patients, the single‐stage stone removal procedure after cannulation times of ≤5, >5 to ≤10, and > 10 to ≤15 min did not increase the incidence of PEP compared to the two‐stage stone removal procedure. However, in all patients and matched patients with cannulation times >15 min, the incidence of PEP was significantly higher in the single‐stage stone removal group than in the two‐stage stone removal group [all patients; 10/41 (24.4%) *vs* 10/119 (8.4%), *P* = 0.013, matched patients; 10/31 (32.3%) *vs* 1/33 (3.0%), *P* = 0.011; Table 5]. The single‐stage stone removal procedure with cannulation times above 15 min was a significant risk factor of PEP as estimated by regression adjustment by propensity score (*P* = 0.014, 95% CI = 1.4–19.4, odds ratio = 5.2; Table 6).

**Table 5:** Cannulation time‐based comparison of the association between the incidence of post‐ERCP pancreatitis and single‐ and two‐stage stone removal procedures

	Incidence of post‐ERCP pancreatitis				
	Single‐stage group		Two‐stage group		
	*n*	%	*n*	%	*P* value
All patients					
Cannulation time ≤ 5 min (*n* = 489)	5	2.9 (5/175)	4	1.3 (4/314)	0.29
5 min < Cannulation time ≤ 10 min (*n* = 159)	3	4.8 (3/63)	4	4.2 (4/96)	1.0
10 min < Cannulation time ≤ 15 min (*n* = 89)	2	7.1 (2/28)	3	4.9 (3/61)	0.65
Cannulation time > 15 min (*n* = 160)	10	24.4 (10/41)	10	8.4 (10/119)	0.013
Matched patients					
Cannulation time ≤ 5 min (*n* = 271)	3	2.1 (3/140)	4	3.1 (4/131)	0.72
5 min < Cannulation time ≤ 10 min (*n* = 89)	1	2.3 (1/43)	1	2.2 (1/46)	1.0
10 min < Cannulation time ≤ 15 min (*n* = 44)	1	5.0 (1/20)	0	0 (0/24)	0.45
Cannulation time > 15 min (*n* = 64)	10	32.3 (10/31)	1	3.0 (1/33)	0.011

ERCP, endoscopic retrograde cholangiopancreatography.

**Table 6:** Effect of single‐stage stone removal procedure compared with the two‐stage one based on the cannulation time for post‐ERCP pancreatitis after regression adjustment by propensity score

	*P* value	95% CI	Odds ratio
Single‐stage stone removal procedure after cannulation time of ≤5 min	0.89	0.24–5.1	1.1
Single‐stage stone removal procedure after cannulation time of >5 to ≤ 10 min	0.86	0.17–8.4	1.2
Single‐stage stone removal procedure after cannulation time of >10 to ≤ 15 min	0.66	0.10–39.1	2.0
Single‐stage stone removal procedure after cannulation time > 15 min	0.014	1.4–19.4	5.2

ERCP, endoscopic retrograde cholangiopancreatography.

### *Efficacy of single‐stage stone removal*

Table 7 shows the number of ERCP attempts for stone clearance and length of hospital stays of matched patients in the single‐ and two‐stage stone removal groups. The mean number of ERCP attempts of complete stone removal was significantly smaller (1.5 attempts *vs* 2.2 attempts, respectively; *P* < 0.001), and the mean length of hospital stays was significantly shorter in the single‐stage stone removal group than in the two‐stage stone removal group (8.6 days *vs* 10.9 days, respectively; *P* < 0.001).

**Table 7:** ERCP sessions for stone clearance and hospital stays of matched patients in the single‐stage and two‐stage stone removal groups

	Single‐stage group	Two‐stage group	*P* value
ERCP sessions (mean [SD])	1.5 (0.64)	2.2 (0.48)	<0.001
Hospital stays (mean [SD], days)	8.6 (5.3)	10.9 (5.6)	<0.001

ERCP, endoscopic retrograde cholangiopancreatography.

## Discussion

This study investigated the incidence of PEP by single‐stage endoscopic stone removal for choledocholithiasis, including asymptomatic CBD stones, elevated liver test results and/or obstructive jaundice without cholangitis, and mild‐to‐moderate acute cholangitis. We conducted a propensity‐matched analysis and regression adjustment by propensity score to adjust for potential confounding factors of PEP. Although single‐stage endoscopic stone removal was not a significant risk factor for PEP overall, this procedure after cannulation times >15 min was a significant risk factor of PEP.

PEP is the most common and serious complication associated with ERCP and can be lethal. A recent systematic review that analyzed 108 randomized controlled studies stated that the PEP incidence was 9.7%, and the PEP mortality was 0.15%.^14^ The risk factor of PEP is divided into patient‐related and procedure‐related risk factors in the available guidelines. Single‐stage endoscopic stone removal is not listed as a risk factor of PEP in the guidelines.^7^, ^15^ However, there are few reports that state whether single‐stage endoscopic stone removal is a risk factor of PEP or not in patients with native papilla.

In the Tokyo guidelines 2018, papillary treatments, such as EST and EPBD, and subsequent single‐stage stone removal are acceptable in patients with mild‐to‐moderate acute cholangitis.^5^ Therefore, single‐stage endoscopic stone removal is often performed for CBD stones in clinical practice. However, we hypothesized that single‐stage stone removal may be a risk factor of PEP because single‐stage endoscopic stone removal in addition to papillary treatment at the same time may exacerbate papillary trauma and lead to pancreatic flow blockage, which is considered to be the cause of PEP.^15^

A retrospective study that included 345 patients with native papilla showed that single‐stage endoscopic stone removal was not a significant risk factor of PEP in multivariate analysis.^8^ However, the aim of the previous study was to examine the overall incidence of complications associated with single‐stage endoscopic stone removal, and the study included biliary pancreatitis. Another study in 50 patients with mild‐to‐moderate acute cholangitis reported that the incidence of ERCP‐related complications was 10% (5/50) and the incidence of PEP was 2% (1/50) after single‐stage endoscopic stone removal procedure.^16^ However, these past reports were not focused on the incidence of PEP arising from single‐stage stone removal.

The present study showed that single‐stage stone removal was not a significant risk factor of PEP after adjusting for potential confounding factors overall. However, in patients who underwent the single‐stage stone removal procedure after cannulation times of >15 min, the incidence of PEP was significantly higher than that in the two‐stage stone removal group. Stone removal subsequent biliary cannulation attempt durations of >15 min can be an invasive procedure that causes exacerbation of papilla trauma sufficient to block pancreatic flow and lead to PEP. The results of the present study suggested that single‐stage stone removal is suitable for patients with cannulation times of ≤15 min.

In this study, the single‐stage stone removal procedure after cannulation time ≤ 15 min was performed in more than 80% of enrolled patients, and this procedure after cannulation time > 15 min was performed in less than 20% of enrolled patients in both all and matched patient groups. There was no statistical difference in the overall incidence of PEP between single‐ and two‐stage stone removal procedures in all and matched patients. This is because the proportion of the single‐stage stone removal procedure after cannulation time ≤ 15 min was greater than that of single‐stage stone removal after cannulation time > 15 min in both all and matched patients.

There are past reports on the devices used to remove CBD stones. Balloon catheters and basket catheters are widely available. Mechanical lithotripsy is performed for cases of difficult stones in which standard stone removal using a balloon or basket catheter cannot be achieved.^17^ A multicenter prospective randomized trial demonstrated that the rate of complete stone clearance with a single catheter for CBD stones of ≤10 mm was higher in the balloon catheter group than in the basket catheter group. One study found that there was no significant difference in the rates of PEP and complete stone clearance in one ERCP session between the balloon catheter group and basket catheter group.^18^ Mechanical lithotripsy achieves successful stone clearance in 80–90% of patients with difficult CBD stones. A prospective study showed that use of a mechanical lithotriptor did not increase the incidence of PEP relative to that occurring after standard sphincterotomy.^19^

The advantages of single‐stage stone removal compared with two‐stage stone removal were found to be the expectation of fewer ERCP attempts and shortened hospital stays, which would lead to reduced medical costs. In the propensity‐matched patients in this study, there were significantly fewer ERCP attempts for stone clearance and hospital stays in the single‐stage stone removal group than in the two‐stage stone removal group.

There were several limitations in the present study. First, although we performed propensity score analysis, including propensity‐matched analysis and regression adjustment by propensity score to adjust for potential confounding factors, some unmeasured confounding factors may not have been excluded. Second, because Kumamoto City Hospital suffered from tremendous damage due to the Kumamoto earthquake in April 2016 and ERCP could not be performed afterward, the data obtained from Kumamoto City Hospital was for 4 years only (April 2012 to April 2016).

In conclusion, we used propensity score analysis to examine the incidence of PEP in single‐stage stone removal for choledocholithiasis with no inflammation or mild‐to‐moderate acute cholangitis. Overall, single‐stage stone removal was not a significant risk factor for PEP. However, single‐stage stone removal procedure after a long cannulation times of >15 min was a significant risk factor of PEP. Single‐stage stone removal may be a safe procedure with respect to PEP in cases with cannulation times of ≤15 min.
